# Inhibitory Effect of a γ-Tocopherol-Rich Mixture of Tocopherols on the Formation and Growth of LNCaP Prostate Tumors in Immunodeficient Mice

**DOI:** 10.3390/cancers3043762

**Published:** 2011-09-28

**Authors:** Xi Zheng, Xiao-Xing Cui, Tin Oo Khor, Ying Huang, Robert S DiPaola, Susan Goodin, Mao-Jung Lee, Chung S Yang, Ah-Ng Kong, Conney Allan H.

**Affiliations:** 1 Susan Lehman Cullman Laboratory for Cancer Research, Department of Chemical Biology, Ernest Mario School of Pharmacy, Rutgers, The State University of New Jersey, 164 Frelinghuysen Road, Piscataway, NJ 08854, USA; E-Mails: xxcui@rci.rutgers.edu (X.-X.C.); maomlee@rci.rutgers.edu (M.J.L.); csyang@rci.rutgers.edu (C.S.Y.); aconney@pharmacy.rutgers.edu (A.H.C.); 2 Cancer Institute of New Jersey, New Brunswick, NJ 08903, USA; E-Mails: dipaolrs@umdnj.edu (R.S.D.); goodin@umdnj.edu (S.G.); kongt@pharmacy.rutgers.edu (A.-N. K.); 3 Department of Pharmaceutics, Ernest Mario School of Pharmacy, Rutgers, The State University of New Jersey, Piscataway, NJ 08854, USA; E-Mails: tkhor@rci.rutgers.edu (T.O.K.); yingh@eden.rutgers.edu (Y.H.)

**Keywords:** prostate cancer, tocopherol, immunodeficient mice, xenograft tumor

## Abstract

In the present study, we determined the effects of a γ-tocopherol-rich mixture of tocopherols (γ-TmT) on the growth and apoptosis of cultured human prostate cancer LNCaP cells. We also determined the effects of dietary γ-TmT on the formation and growth of LNCaP tumors in immunodeficient mice. In the *in vitro* study, we found that the activity of γ-TmT was stronger than α-tocopherol for inhibiting the growth and stimulating apoptosis in LNCaP cells. In the animal study, treatment of severe combined immunodeficient (SCID) mice with dietary γ-TmT inhibited the formation and growth of LNCaP xenograft tumors in a dose-dependent manner. Mechanistic studies showed that γ-TmT administration inhibited proliferation as reflected by decreased mitosis and stimulated apoptosis as reflected by increased caspase-3 (active form) expression in LNCaP tumors. In addition, dietary administration of γ-TmT increased the levels of α-, γ- and δ- tocopherol in plasma, and increased levels of γ- and δ- tocopherol were also observed in the prostate and in tumors. The present study demonstrated that γ-TmT had strong anticancer activity both *in vitro* and *in vivo*. Additional studies are needed to determine the potential preventive effect of γ-TmT for prostate cancer in humans.

## 1. Introduction

Vitamin E exists in eight different forms that include four tocopherols and four tocotrienols. Tocopherols are plant-derived polyphenolic compounds. Depending upon the number and position of methyl groups on the chromanol ring, they exist as α-, β-, γ- or δ-tocopherol [1,2]. Among them, α-tocopherol is the predominant form of vitamin E in plasma and tissues and is the form that has drawn the most attention. Several epidemiological studies have shown that a lower vitamin E nutritional status is associated with increased risk of certain types of cancers [3-7], but the results from epidemiological studies on the protective role of tocopherols against prostate cancer are inconsistent [3]. Although a previous clinical trial using α-tocopherol and β-carotene showed that fewer cases of prostate cancer were diagnosed among those who received α-tocopherol than among those who did not [8], the results of a large-scale human trial using α-tocopherol and selenium failed to show a preventive effect on prostate cancer [9]. A possible explanation for the negative outcome of this study was that α-tocopherol may not have been the right form of tocopherol for the prevention of prostate cancer. Many studies indicate that γ-tocopherol, the major form of vitamin E in U.S. diets, has more potent antiinflammatory and anticancer activities than α-tocopherol [10-15]. γ-Tocopherol has been shown to induce apoptosis in cultured prostate cancer cells [16]. Recent studies from our research team at Rutgers University showed that a γ-tocopherol-rich mixture of tocopherols (γ-TmT; containing 13% α-tocopherol, 1.5% β-tocopherol, 57% γ-tocopherol and 24% δ-tocopherol) inhibited carcinogenesis in a number of animal models [17-20].

We hypothesize that γ-TmT has a stronger inhibitory effect on the growth of cultured prostate cancer cells than α-tocopherol, and that γ-TmT will strongly inhibit the growth of prostate xenograft tumors in immunodeficient mice. To test these hypotheses, we compared the effects of α-tocopherol and γ-TmT on growth and apoptosis in cultured human prostate cancer LNCaP cells. We also determined the effects of γ-TmT on the formation and growth of xenograft LNCaP tumors in severe combined immunodeficient (SCID) mice. In the *in vitro* study, we found that γ-TmT had stronger effects for inhibiting the growth and stimulating apoptosis than α-tocopherol in LNCaP cells. We also found that administration of γ-TmT strongly inhibited the formation and growth of LNCaP tumors in SCID mice.

## 2. Results and Discussion

### 2.1. Effects of α-tocopherol and γ-TmT on Growth and Apoptosis in Cultured LNCaP Cells

**Figure 1.**
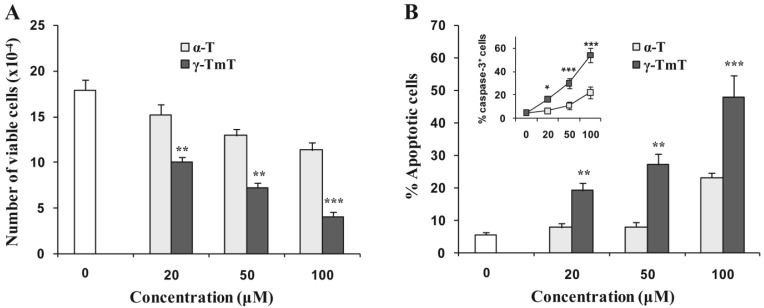
Effects of γ-TmT and α-tocopherol on growth and apoptosis in cultured LNCaP cells. LNCaP cells were seeded at a density of 5 × 10^4^ cells/ml in RPMI medium supplemented with 10% FBS. After 24 h incubation, the medium was changed to RPMI supplemented with 1% FBS and the cells were treated with α-tocopherol or γ-TmT at concentrations of 20, 50 and 100 μM for 96 h. (**A**) Number of viable cells after treatment as determined by a trypan blue exclusion assay. The number of apoptotic cells was determined by morphological assessment (**B**) and caspase-3 (active form) assay (B insert). Differences for the number of viable cells between the α-tocopherol-treated group and the γ-TmT-treated group at different concentrations were analyzed by the Tukey-Kramer Multiple Comparison Test ** p <0.01, *** p < 0.001.

The effects of α-tocopherol and γ-TmT on the growth and apoptosis of cultured prostate cancer LNCaP cells were determined. LNCaP cells were treated with different concentrations of α-tocopherol or γ-TmT for 96 h. The molar concentration of γ-TmT was assessed by adding the molar concentrations of the four forms of tocopherols determined by their percentages in the mixture. The number of viable cells was determined by the trypan blue exclusion assay, and apoptosis was determined by morphological assessment. Treatment of LNCaP cells with α-tocopherol or γ-TmT resulted in a concentration-dependent decrease in the number of viable cells (Figure 1A).

As shown in Figure 1A, γ-TmT had a stronger inhibitory effect on the growth of LNCaP cells than α-tocopherol. Treatment of LNCaP cells with α-tocopherol or γ-TmT also caused an increase in apoptosis. As shown in Figure 1B, γ-TmT had a stronger stimulatory effect on apoptosis in LNCaP cells than α-tocopherol. When LNCaP cells were treated with 20 μM γ-TmT (containing approximately 11.4 μM γ-tocopherol, 4.8 μM δ-tocopherol and 2.6 μM α-tocopherol), we observed a 44% decrease in the number of viable cells and a 242% increase in apoptotic cells (Figure 1). Since α-tocopherol at 20 μM only caused a 15% decrease in the number of viable cells and a 39% increase in apoptotic cells, the effects on growth inhibition and apoptosis observed in the cells treated with 20 μM γ-TmT are likely caused by γ-tocopherol, δ-tocopherol or the combination of different tocopherols in γ-TmT. In our preliminary *in vitro* experiment, we found that δ- and γ-tocopherol was more active than α-tocopherol for inhibiting the growth of human prostate cancer LNCaP cells (data not shown). It has been shown that γ-tocopherol or the combination of γ- and δ-tocopherol were more effective than α-tocopherol for inhibiting the growth of several types of cancer cells in culture [15,16,21-23]. In addition, our recent study demonstrated that δ- and γ-tocopherol were more active than α-tocopherol for inhibiting lung tumorigenesis [24]. Earlier studies suggested that mixtures of tocopherols are superior to a single tocopherol for inhibiting inflammation [25,26]. Studies from our collaborative research team have shown that γ-TmT has stronger anticancer activity than α-tocopherol in several types of cancer cells [17-20]. In line with these results, our present study indicates that the γ-TmT has stronger effects than α-tocopherol for inhibiting growth and inducing apoptosis in cultured LNCaP cells.

### 2.2. Inhibitory Effect of Dietary γ-TmT on the Formation and Growth of LNCaP Tumors in SCID Mice

**Figure 2.**
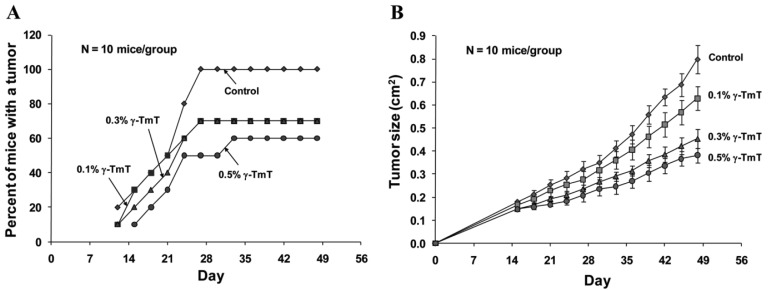
Inhibitory effect of dietary γ-TmT on the formation and growth of prostate LNCaP xenograft tumors in SCID mice. Male SCID mice were injected subcutaneously with 2.0 × 10^6^ cells/mouse of LNCaP cells in Matrigel and treated with 0.1, 0.3 or 0.5% dietary γ-TmT for 48 days. Each group had 10 mice. (**A**) Percentage of animals that formed LNCaP tumor. (**B**) The average tumor size (length × width; cm^2^) in each group was measured every third day. Each value represents the mean ± S.E.

Because γ-TmT showed strong effects on growth inhibition and apoptosis in cultured LNCaP cells, we further investigated the effect of this tocopherol mixture on the formation and growth of LNCaP tumors in immunodeficient SCID mice. We found that treatment of the mice with dietary γ-TmT inhibited the formation of LNCaP tumors in a dose-dependent manner (Figure 2A). At the end of the experiment, all animals in the control group developed tumors, 70% of mice in the 0.1% γ-TmT-treated or 0.3% γ-TmT-treated group developed tumors, and only 60% of the mice in the 0.5% γ-TmT-treated group developed tumors. These results indicate that γ-TmT has a preventive effect on the formation of prostate cancer in a mouse xenograft model.

Treatment with γ-TmT also inhibited the growth of LNCaP tumors in the mice in a dose-dependent manner (Figure 2B). At the end of the experiment, the average tumor size ± S.E as determined by measuring the length and width of each tumor (cm^2^) was 0.80 ± 0.06 in the control group, 0.63 ± 0.05 in the 0.1% γ-TmT-treated group, 0.45 ± 0.04 in the 0.3% γ-TmT-treated group and 0.38 ± 0.03 in the 0.5% γ-TmT-treated group. Statistical analysis using ANOVA with Tukey-Kramer multiple comparison test showed that the differences for average tumor size between the control group and the 0.3% γ-TmT-treated group, and between the control group and the 0.5% γ-TmT-treated group were statistically significant (p < 0.001). These results indicate that γ-TmT dose-dependently inhibits the growth of LNCaP tumors in the mice. To our knowledge, this is the first report for an inhibitory effect of a mixed tocopherol preparation on the formation and growth of human prostate tumors in a xenograft model. A recent study from our collaboration team showed that γ- or δ-tocopherol was more active than α-tocopherol in inhibiting lung tumorigenesis *in vivo* [24]. Future studies are needed to determine the anti-carcinogenic effect of γ- and δ-tocopherol in suitable prostate cancer models.

In the present study, we found that treatment with dietary γ-TmT had no effect on the body weight of the mice. The mean ± S.E for the percent of initial body weight after 48 days of treatment was 87.6 ± 5.4 for the control group, 85.4 ± 4.3 for the 0.1% γ-TmT-treated group, 82 ± 5.2 for the 0.3% γ-TmT-treated group and 90.3 ± 5.4 for the 0.5% γ-TmT-treated group. Statistical analysis using ANOVA with the Tukey-Kramer multiple comparison test showed that the difference in percent of initial body weight between any two groups was not statistically significant (p > 0.05).

### 2.3. Inhibitory Effect of γ-TmT on Mitosis and Apoptosis in LNCaP Tumors

The effects of treatment with γ-TmT for 48 days on proliferation and apoptosis in LNCaP tumors described in Figure 2 were studied by determining mitotic cells and caspase-3 (active form) positive cells in these tumors. As shown in Table 1, the percent of mitotic cells was decreased in LNCaP tumors from mice treated with γ-TmT in a dose-dependent manner. Apoptosis as measured by the percent of caspase-3 (active form) positive cells in LNCaP tumors was increased in LNCaP tumors from mice treated with γ-TmT (Table 1). The ratio of the percent mitotic cells/percent caspase-3 (active form) positive cells in LNCaP tumors was also decreased in a dose-dependent manner (Table 1). These results indicate that the strong inhibitory effect of γ-TmT on the growth of LNCaP tumors in SCID mice may be mediated by inhibition of proliferation and stimulation of apoptosis in the tumors. The inhibitory effects of γ-TmT on the formation and growth of LNCaP tumors in SCID mice may be mediated by other mechanisms such as suppression of angiogenesis. Future studies are needed to determine the effect of γ-TmT on vascularization of the tumors.

### 2.4. Plasma and Tissue Levels of α-, γ- and δ-Tocopherol in SCID Mice Treated with γ-TmT for 48 Days

In our study, SCID mice were fed AIN 93M diet containing 0, 0.1, 0.3 or 0.5% γ-TmT for 48 days. The plasma concentrations of α-, γ- and δ-tocopherol in the control animals fed AIN 93M diet without γ-TmT were low (Table 2). Treatment of the mice with dietary γ-TmT increased the plasma levels of α-, γ- and δ-tocopherol. As shown in Table 2, treatment of the mice with γ-TmT did not increase the levels of α-T in the prostate and tumors. Treatment of the mice with 0.3% γ-TmT in the diet increased γ-tocopherol levels by 6.1- and 1.2-fold in the prostate and tumors, respectively, and this treatment increased the level of δ-tocopherol by 7.0- and 0.9-fold in the prostate and tumors, respectively. Further increases in α-, γ- and δ-tocopherol were not observed in mice fed 0.5% γ-TmT diet. A possible explanation is that a high dose of γ-TmT induced enzymes that metabolize the tocopherols. Further studies are needed to determine the level of metabolites of α-, γ- and δ-tocopherol in the prostate and tumors from mice fed 0.5% γ-TmT diet. Mice treated with up to 0.5% γ-TmT in the diet did not have an effect on body weight. The mean ± SE serum levels of α-tocopherol and γ-tocopherol in humans were 30.09 ± 0.45 μmol/mL and 5.74 ± 0.22 μmol/mL, respectively [27]. The doses of γ-TmT used in the present *in vitro* and *in vivo* studies were chosen based on the effective doses of γ-TmT in our previous studies using cell culture and various animal models [17-20]. Although the plasma levels of different tocopherols were much lower than the concentrations needed for these tocopherols to inhibit growth and induce apoptosis *in vitro*, we observed a strong inhibitory effect of γ-TmT on the formation and growth of LNCaP tumors in SCID mice. A possible explanation is that the drug effects were cumulative in the animals after they were treated with γ-TmT for a long time period. Another possible explanation is that the different tocopherols or their metabolites may act together with some endogenous substances and enhance the effect on growth inhibition and apoptosis induction. In our planned phase II clinical trial in prostate cancer patients, the doses of commercial γ-TmT (Advanced High Gamma Vitamin E; Nature's Bounty, Inc. Bohemia, NY, USA) are 400 mg (1 capsule) or 800 mg (2 capsules) per day which are higher than physiological doses.

## 3. Experimental Section

### 3.1. Cells and Reagents

LNCaP cells were obtained from the American Type Culture Collection (ATCC, Rockville, MD, USA). α-tocopherol was from Sigma-Aldrich (St. Louis, MO, USA). γ -TmT was from Cognis Corp. (Cincinnati, OH, USA). Matrigel was obtained from BD Biosciences (Bedford, MA). RPMI-1640 tissue culture medium, penicillin-streptomycin, L-glutamine and fetal bovine serum (FBS) were from Gibco (Grand Island, NY, USA). LNCaP cells were maintained in RPMI-1640 culture medium containing 10% FBS that was supplemented with penicillin (100 units/mL)-streptomycin (100 μg/mL) and L-glutamine (300 μg/mL). Cultured cells were grown at 37 °C in a humidified atmosphere of 5% CO_2_ and were passaged twice a week. Proliferating LNCaP cells at about 70% confluence were used for the animal experiment as indicated below. α-tocopherol and γ-TmT were dissolved in DMSO. The molar concentration of the tocopherols in γ-TmT was calculated from the sum of 13% α-tocopherol, 1.5% β-tocopherol, 57% γ-tocopherol and 24% δ-tocopherol in the solution. The final concentration of DMSO in the culture medium was 0.1%.

### 3.2. Determination of the Number of Viable Cells

The number of viable cells after each treatment was determined using a hemacytometer under a light microscope (Nikon Optiphot, Japan). Cell viability was determined by the trypan blue exclusion assay, which was done by mixing 80 μL of cell suspension and 20 μL of 0.4% trypan blue solution for 2 min. Blue cells were counted as dead cells and the cells that did not absorb dye were counted as live cells.

### 3.3. Morphological Assessment of Apoptotic Cells

Apoptosis was determined by morphological assessment in cells stained with propidium iodide [28-30]. Briefly, cytospin slides were prepared after each experiment and cells were fixed with acetone/methanol (1:1) for 10 min at room temperature, followed by 10 min propidium iodide staining (1 μg/mL in PBS) and analyzed using a fluorescence microscope (Nikon Eclipse TE200, Japan). Apoptotic cells were identified by classical morphological features including nuclear condensation, cell shrinkage, and formation of apoptotic bodies as we have done previously [28-30].

### 3.4. Xenograft LNCaP Tumors in Immunodeficient Mice

Male SCID mice were obtained from Taconic Farms Inc. (Germantown, NY, USA). The animals were housed in sterile filter-capped microisolator cages. The γ-TmT used was a mixture containing 57% γ-tocopherol, 24% δ- tocopherol, 13% α- tocopherol and 1.5% β- tocopherol.

LNCaP cells (2.5 × 10^6^ cells/0.1 mL/mouse) suspended in 50% Matrigel (BD Biosciences, Bedford, MA). in RPMI 1640 medium were injected subcutaneously into the right flank of the mice [31]. Immediately after the tumor cell injection, the mice were randomly assigned to 4 groups. Mice in group 1 were fed AIN 93M diet, mice in group 2 were fed AIN 93M diet containing 0.1% γ-TmT, mice in group 3 were fed AIN 93M diet containing 0.3% γ-TmT, and mice in group 4 were fed AIN 93M diet containing 0.5% γ-TmT. Each group had 10 mice. The mice were given these experimental diets for 48 days. Once a palpable tumor was formed, tumor size (length × width; cm^2^) was measured once every third day. The animal experiment was carried out under an Institutional Animal Care and Use Committee (IACUC)-approved protocol.

### 3.5. Determination of Mitotic Cells

LNCaP tumors in mice from each experimental group were excised. The samples were fixed overnight in 10% formalin and then transferred into 70% ethanol. Paraffin-embedded tissue sections of 5-μm thickness were stained with hematoxylin and eosin (H&E). Mitotic cells were counted under a light microscope as described elsewhere [32].

### 3.6. Caspase-3 (active form) Immunostaining

An antibody that reacts with the active form of caspase-3 was purchased from R&D Systems (Minneapolis, MN, Catalog number: AF835). Tumor sections used for the measurement of caspase-3 (active form) were stained by the horseradish peroxidase-conjugated avidin method with some modification [33]. Briefly, sections were incubated with caspase-3 primary antibody (1:2,000 dilution) for 30 min at room temperature followed by incubation with a biotinylated anti-rabbit secondary antibody for 30 min and incubation with conjugated-avidin solution (ABC ellite kit purchased from Vector Laboratories, Burlingame, CA) for 30 min. Color development was achieved by incubation with 0.02% 3,3′-diaminobenzidine tetrahydrochloride containing 0.02% hydrogen peroxide for 10 min at room temperature. The slides were then counterstained with hematoxylin, dehydrated, and coverslips were added for permanent mounting. A positive reaction was shown as a brown precipitate in the cytoplasm and/or perinuclei of the cells. The percent of caspase-3 positive cells was determined in each tumor.

### 3.7. Analysis of Tocopherols by High-Performance Liquid Chromatography

Our previous procedure was used for the determination of tocopherol levels [17-20]. In brief, fat-soluble substances in plasma or tumor samples were extracted with hexane, redissolved in ethanol and then analyzed by high performance liquid chromatography. The tocopherol concentrations in mouse plasma and tissues were determined by comparison with the peak heights of standards that were added to plasma.

### 3.8. Statistical Analyses

The analysis of variance (ANOVA) with Tukey-Kramer multiple comparison test was used for the comparison of growth inhibition and apoptosis in cultured LNCaP cells treated with different tocopherols, and for comparison of tumor size and body weight in different groups of animals at the end of the experiment.

## 4. Conclusions

In the present study, we demonstrated that a γ-tocopherol-rich mixture of tocopherols, γ-TmT, was more potent than α-tocopherol for inhibiting the growth and stimulating apoptosis in cultured human prostate cancer LNCaP cells. We also demonstrated that dietary γ-TmT dose-dependently inhibited the formation and growth of LNCaP tumors in SCID mice. Gamma-TmT is commercially available as “Advanced High Gamma Vitamin E” (Nature's Bounty, Inc. Bohemia, NY, USA). Since our study demonstrated a strong inhibitory effect of γ-TmT on the growth of LNCaP tumors in mice without apparent toxicity, clinical studies for determining the potential preventive/therapeutic effect of this agent in individuals with a high risk of prostate cancer or in early stage prostate cancer patients are warranted.

## Figures and Tables

**Table 1.** Effects of dietary γ-TmT on the percentage of mitotic and caspase-3 positive cells in LNCaP tumors.

**Treatment**	**No. of tumors**	**Percent mitotic cells**	**Percent caspase-3 positive cells**	**Ratio of percent mitotic cells/caspase-3 positive cells**
Control	10	0.59 ± 0.03	0.34 ± 0.02	1.81 ± 0.11
0.1% γ-TmT	7	0.56 ± 0.03	0.40 ± 0.02	1.44 ± 0.08
0.3% γ-TmT	7	0.38 ± 0.02 **	0.47 ± 0.03 *	0.86 ± 0.10 **
0.5% γ-TmT	6	0.33 ± 0.02 **	0.48 ± 0.03 *	0.76 ± 0.12 **

Male SCID mice were injected subcutaneously with 2.0 × 10^6^ LNCaP cells/mouse and treated with 0.1, 0.3 or 0.5% dietary γ-TmT for 48 days. LNCaP tumors from mice at the end of the experiment were analyzed for mitotic and caspase-3 (active form) positive cells. Mitotic cells were identified and counted in H&E stained tissue sections using a light microscope. Caspase-3 positive cells were identified by immunostaining. Each value represents the mean ± S.E.

* p < 0.05,

** p < 0.001 as compared with control.

**Table 2.** Levels of different tocopherols (T) in plasma, prostate and tumors from SCID mice treated with dietary γ-TmT.

**Treatment**	**Plasma (μM ± SE)**			**Prostate (μmol/kg ± SE)**			**Tumor (μmol/kg ± SE)**		

	**α-T**	**γ-T**	**δ-T**	**α-T**	**γ-T**	**δ-T**	**α-T**	**γ-T**	**δ-T**
Control	0.73 ± 0.08	0.61 ± 0.09	0.11 ± 0.02	3.76 ± 1.14	0.13 ± 0.05	0.06 ± 0.03	2.95 ± 0.26	0.29 ± 0.01	0.19 ± 0.01
0.1% γ-TmT	3.80 ± 1.41	0.71 ± 0.09	0.13 ± 0.01	3.37 ± 0.84	0.33 ± 0.06	0.19 ± 0.03	3.10 ± 0.50	0.65 ± 0.03	0.37 ± 0.02
0.3% γ-TmT	2.87 ± 1.08	0.99 ± 0.14	0.18 ± 0.03	4.50 ± 0.84	0.92 ± 0.19	0.48 ± 0.11	3.26 ± 1.02	0.64 ± 0.17	0.36 ± 0.07
0.5% γ-TmT	5.55 ± 1.57	1.18 ± 0.24	0.23 ± 0.06	2.57 ± 0.74	0.63 ± 0.16	0.41 ± 0.09	2.16 ± 0.57	0.33 ± 0.03	0.22 ± 0.01

Plasma, prostate and tumors from the experiment described in Figure 1 were collected for analysis of different tocopherols (T). Levels of α-T, γ-T and δ-T were determined according to the Materials and Methods. The detection limit of each tocopherol in our analysis was 0.02 ng.
